# Association between H19 SNP rs217727 and lung cancer risk in a Chinese population: a case control study

**DOI:** 10.1186/s12881-018-0573-1

**Published:** 2018-08-02

**Authors:** Lingling Li, Genyan Guo, Haibo Zhang, Baosen Zhou, Lu Bai, He Chen, Yuxia Zhao, Ying Yan

**Affiliations:** 1 Department of Radiotherapy Oncology, The Fourth Affiliated Hospital of China Medical University, No.4 Chongshan East Road, Huanggu District, Shenyang, Liaoning 110032 People’s Republic of China; 2 Department of Radiation Oncology, The General Hospital of Shenyang Military Command, No.83 Wenhua Road, Shenhe District, Shenyang, Liaoning 110016 People’s Republic of China; 3 Department of Epidemiology, China Medical University, Shenyang, Liaoning China; 4 Department of Radiotherapy Oncology, The First Affiliated Hospital of China Medical University, Shenyang, Liaoning China

**Keywords:** Lung cancer, H19, Susceptibility, SNP

## Abstract

**Background:**

H19 was the first long non-coding RNA (lncRNA) to be confirmed. Recently, studies have suggested that H19 may participate in lung cancer (LC) development and progression. This study assessed whether single nucleotide polymorphisms (SNPs) in H19 are associated with the risk of LC in a Chinese population.

**Methods:**

A case-control study was performed, and H19 SNP rs217727 was analyzed in 555 lung cancer patients from two hospitals and 618 healthy controls to test the association between this SNP and the susceptibility to LC.

**Results:**

The A/A homozygous genotype of rs217727 was significantly associated with an increased LC risk (odds ratio (OR) = 1.661, 95% confidence interval (CI) = 1.155 to 2.388, *P* = 0.006). Significant associations remained after stratification by smoking status (*P* < 0.001). Furthermore, the A/A genotype had a higher risk of LC than those of G/G in the squamous cell carcinoma (OR = 2.022, *P* = 0.004) and adenocarcinoma (OR = 1.606, *P* = 0.045) subgroups.

**Conclusions:**

The rs217727 SNP in lncRNA H19 was significantly associated with susceptibility to LC, particularly in squamous cell carcinoma and adenocarcinoma, and identified the homozygous A/A genotype as a risk factor for LC.

## Background

Lung cancer (LC) has a high incidence and will continue to be the most common cause of cancer-related death around the world [1]. In China, this malignancy has the highest mortality and accounts for an estimated 25% of cancer-related deaths [2]. LC is a complex pathological process. The major risk factors by far are cigarette smoking and air pollution. Because a proportion of individuals exposed to carcinogens may have genetic factors associated with the development of cancer, predisposing genic elements should be weighed as risk factors for LC.

Long non-coding RNAs (lncRNAs) are longer than 200 nucleotides and are defined as non-protein-coding transcripts that are universally transcribed in the genome [3]. LncRNAs are transcribed as sense, antisense, bidirectional, intronic, or intergenic [4]. They can work by binding to chromatin-modifying complexes to specifically silence genomic loci both in cis and trans [5]. Increasingly, more studies have revealed that lncRNAs play a major role in many aspects of tumorigenesis at the epigenetic, transcriptional, and posttranscriptional levels, including cell growth, apoptosis, invasion, and metastasis. Based on the latest studies, there is evidence that lncRNAs can control gene expression through multiple mechanisms, such as transcription, translation, imprinting, genome rearrangement, and chromatin modification [6]. H19 is a maternally expressed imprinted gene on chromosome 11p15.5 that encodes for a capped and spliced RNA and has been implicated in cancer [7]. It was the first lncRNA discovered in the human genome and plays a crucial role in mammalian development [8, 9].

Single nucleotide polymorphisms (SNPs) have been widely used in plant, livestock, and animal genetic analyses. SNPs may affect gene expression and function. In addition, SNPs can be associated with the susceptibility to cancer. To date, there have been rare reports of genetic mutations in lncRNAs and their possible correlations to LC susceptibility. Thus far, the association between H19_rs217727 polymorphisms and LC has not been studied in the Chinese population.

In this hospital-based case-control study, we hypothesized a possible association between variant genotypes of the human H19 gene (rs217727) and LC. To test our hypothesis, SNPs within the H19 gene were genotyped from blood DNA samples of 555 LC patients and 618 age- and gender-matched general population controls.

## Methods

### Study population

The study population consisted of 555 LC patients and 618 healthy controls. The LC patients were consecutively recruited between September 2010 and November 2015 from the First Affiliated Hospital of China Medical University and the Fourth Affiliated Hospital of China Medical University. Each patient was histopathologically diagnosed including squamous cell carcinoma (SCC), adenocarcinoma (AD) and small cell lung cancer (SCLC). These control subjects were picked out throughout the same period in the Fourth Affiliated Hospital of China Medical University from the health examination center. Allowing for a better condition, the following exclusion criteria were used: history of LC; history of significant concomitant tumors; any cancer-related metastasis; chemotherapy or radiotherapy; non autologous transfusion. All subjects (LC patients and healthy controls) participated had no family history of LC in this study. Then, we randomly took sample of 618 healthy controls, which were frequency matched to the LC cases on age and gender. All participants who were unrelated ethnic Chinese resided in or near Liaoning province. All individual participants voluntarily joined this study with informed consents. Information was collected by a structured questionnaire. Smoking was defined as ≥10 cigarettes per day for at least 2 years.

### SNPs selection and genotyping

The location of the 2.7 kb human H19 gene (Gene ID: 283120) including the DMR (differentially methylated regions) and the promoter region was pinpointed to chromosome 11, position (1972982–1981641). The HapMap project has established a common pattern in the human genome for most of the population on the basis of DNA sequence variation. Based on the HapMap data and the criteria of minor allele frequency (MAF) > 0.05 in CB population, we found two SNPs rs217727 and rs2107425 in H19, and they are in high linkage disequilibrium (LD). Some researchers had found that H19_rs2107425 and H19_rs217727 play roles in carcinoma susceptibility. The role of rs2107425 polymorphism had been identified in lung cancer. So, we chose the other one SNP, rs217727.

Genomic DNA was extracted from venous blood. Usually, about 5 ml venous blood samples were collected from each participant. The blood samples are registered and stored at − 80 °C. Genomic DNA was extracted from leukocytes, and separated from the whole blood using a standard phenol-chloroform protocol. Genotyping was performed by pre-designed TaqMan probes (Applied Biosystems, Foster City, CA, USA). The assay ID is C___2603707_10 (part number: 4351379), and the specific amplicon context sequence is TGTGGTGGCTGGTGGTCAACCGTCC[A/G]CCGCAGGGGGTGGCCATGAAGATGG (Table 1) The H19_rs217727 polymorphism was amplified and genotyped through the TaqMan SNP Genotyping Assay by using the ABI 7500 Real-time PCR system (Applied Biosystems, Foster City, CA, USA) in 96-well plates. The reaction mixture (5 μl) contained 2.5 μl TaqMan® Genotyping Master Mix (Applied Biosystems, Foster City, CA, USA), 0.125 μl hydrolysis probe, 1.375 μl ddH2O and 30 ng genomic DNA for each SNP, according to the following PCR protocol: 95 °C for 10 min for 1 cycle; 95 °C for 15 s and 60 °C for 1 min for 40 cycles; followed by a cycle of 60 °C for 1 min which is a stage of analysis for genotypes. Controls (known genotype and water) were included in each reaction plate to ensure that the genotyping were accuracy. The deionized water was used as a negative control and the rs3219073/GG SNP of PARP-1 was used as a positive control, which was previously detected in many lung cancer samples [10]. Two researchers analyses the genotype individually in a blind method. Approximately 10% samples were randomly selected to repeat detection, the results for random sampling were 100% concordant as quality control samples.

**Table 1:** Genotyped SNP ordered according to the position in the H19 gene

dbSNP	NCBI assembly location (Build 37)	TaqMan assay ID	Base change	Tag SNP (CHB population; HapMap)	Cinfirmed functional effect of SNP
rs217727	Chr.11:2016908	C___2603707_10	A/G	Yes	No
Context Sequence: TGTGGTGGCTGGTGGTCAACCGTCC[A/G]CCGCAGGGGGTGGCCATGAAGATGG					

### Statistical analysis

The data obtained were computed and analyzed via SPSS, version 16.0 (SPSS Inc., Chicago, IL, USA). Continuous variables without skewness were estimated via means ± standard derivation (SD) and compared with the Student’s t tests. Categorical variables were used through frequency counts and compared by the Chi-square (χ^2^) test [11]. The Hardy–Weinberg equilibrium (HWE) was estimated by the goodness-of-χ^2^ test. When the HWE was respected, the allele comparison and the additive model were asymptotically equivalent [12]. Correlations between the genotype and the susceptibility of LC were assessed via odds ratio (OR) with 95% confidence interval (CI) by logistic regression analyses with adjustment for age and smoking status [13]. OR was also evaluated subgroup, viz. tumors of different pathological types. A value of *P* < 0.05 was considered statistically significant.

## Results

### Characteristics of the study population

The demographics of the 555 LC patients and 618 healthy controls are summarized in Table 2. The majority of the LC patients were diagnosed with adenocarcinoma (44.6%) followed by squamous cell carcinoma (38.7%) and small cell carcinoma (16.7%). The mean ages of the LC patients and healthy controls were 60.15 ± 9.896 and 60.05 ± 10.170 years, respectively. There was no significant difference in the frequency distributions of age or gender (*P* = 0.517 and 0.798) between the LC patients and the controls. However, the data was significantly higher (61.8%) in cases with the smoking status than that in controls (*P* < 0.001), which is consistent with the epidemiological distribution of LC.

**Table 2:** Baseline characteristics of study subjects

Characteristics	Case (*n* = 555)	Controls (*n* = 618)	*P* value
Age (year)	60.15 ± 9.896	60.05 ± 10.170	0.517
Sex			0.798
Male (%)	394 (70.1)	434 (70.2)	
Female (%)	161 (29.9)	184 (29.8)	
Smoking status			< 0.001
Ever (%)	343 (61.8)	143 (23.1)	
Never (%)	212 (38.2)	475 (76.9)	
Pathological types			–
Squamous Cells Carcinoma (%)	215 (38.7)	–	
Adenocarcinoma (%)	248 (44.6)	–	
Small Cell Carcinoma (%)	92 (16.7)	–	

### H19 polymorphisms and the susceptibility of LC

The genotype of H19_rs217727 and its association with the risk of LC are presented in Table 3. The genotype distribution of the rs217727 in the controls did not deviate from those expected under HWE (*P* = 0.167). A statistically significant increase in the risk of LC was found for carriers of the A/A genotype compared to the homozygous carriers of the wild-type G/G genotype (OR = 1.661, 95%CI = 1.155–2.388, *P* = 0.006). After adjustment for the smoking status, the A/A genotype was also significant (*P* = 0.002). However, when the combined A/G + A/A genotypes were compared to the wild-type G/G genotype, there was no significant difference.

**Table 3:** H19 polymorphisms (rs217727) among the cases and controls and the associations with risk of LC

Genotype	Case, *n* (%)	Control, *n* (%)	Crude OR, (95%CI)	*P* value	Adjusted OR, (95%CI)^a^	*P*^a^ value
G/G	210 (37.9)	246 (39.8)	1.0(ref.)		1.0(ref.)	
A/G	250 (45.0)	305 (49.4)	0.960 (0.749~ 1.231)	0.749	0.957 (0.730~ 1.255)	0.751
A/A	95 (17.1)	67 (10.8)	**1.661 (1.155~ 2.388)**	**0.006**	**1.849 (1.248~ 2.740)**	**0.002**
A/G + A/A	345 (62.1)	372 (60.2)	1.086 (0.859~ 1.375)	0.490	1.111 (0.860~ 1.435)	0.420

The bold values mean statistically significance with *P* < 0.05

*LC* lung cancer, *OR* odds ratio, *CI* confidence interval

^a^Adjusted for smoking status

### Stratified analysis of H19 polymorphisms and the risk of LC

We carried out stratified analysis to assess the relationship between the H19 lncRNA SNPs and the risk of LC according to the pathological subtypes (Table 4). We found that the rs217727 A/A genotype was associated with an increased cancer risk for squamous cell carcinoma (*P* = 0.004, adjusted OR = 1.996, 95% CI = 1.142 to 3.489, *P* = 0.015) and adenocarcinoma (*P* = 0.045, adjusted OR = 1.767, 95% CI = 1.096 to 2.850, *P* = 0.019), but not for small cell carcinoma (*P* = 0.123, adjusted OR = 1.799, 95% CI = 0.846 to 3.827, *P* = 0.127). There was no significant association between this polymorphism and the susceptibility of LC with other genotypes.

**Table 4:** Stratification analyses of H19 polymorphisms (rs217727) and risk of LC

Pathological types	Case, *n* (%)	Control, *n* (%)	Crude OR, (95%CI)	*P* value	Adjusted OR, (95%CI)^a^	*P*^a^ value
Squamous Cells Carcinoma						
G/G	81 (37.7)	217 (41.6)	1.0 (ref.)		1.0 (ref.)	
A/G	94 (43.7)	252 (48.3)	0.999 (0.705~ 1.416)	0.997	0.790 (0.532~ 1.174)	0.244
**A/A**	**40 (18.6)**	**53 (10.1)**	**2.022 (1.247~ 3.279)**	**0.004**	**1.996 (1.142~ 3.489)**	**0.015**
A/G + A/A	134 (62.3)	305 (58.4)	1.177 (0.849~ 1.631)	0.327	0.977 (0.675~ 1.414)	0.903
Adenocarcinoma						
G/G	97 (39.1)	228 (40.1)	1.0 (ref.)		1.0 (ref.)	
A/G	110 (44.4)	281 (49.4)	0.920 (0.665~ 1.272)	0.615	0.904 (0.647~ 1.262)	0.552
**A/A**	**41 (16.5)**	**60 (10.5)**	**1.606 (1.011~ 2.551)**	**0.045**	**1.767 (1.096~ 2.850)**	**0.019**
A/G + A/A	151 (60.9)	341 (59.9)	1.041 (0.767~ 1.412)	0.797	1.047 (0.765~ 1.433)	0.776
Small Cell Carcinoma						
G/G	32 (34.8)	187 (41.6)	1.0 (ref.)		1.0 (ref.)	
A/G	46 (50.0)	216 (48)	1.245 (0.761~ 2.035)	0.383	1.114 (0.680~ 1.926)	0.612
A/A	14 (15.2)	47 (10.4)	1.741 (0.860~ 3.522)	0.123	1.799 (0.846~ 3.827)	0.127
A/G + A/A	60 (65.2)	263 (58.4)	1.333 (0.835~ 2.129)	0.229	1.253 (0.764~ 2.056)	0.372

The bold values indicate statistical significance (*P* < 0.05)

*LC* lung cancer, *OR* odds ratio, *CI* confidence interval

^a^ Adjusted for smoking status

## Discussion

LncRNAs, which characterize a functionally varied class of transcripts, have been found in many different species, such as humans, animals, plants, yeast, and viruses [14–17]. Many researchers suggest that lncRNAs play a key role in tumorigenesis and during cellular development, differentiation, and many other biological processes. Furthermore, several studies have reported that lncRNAs are misregulated in various types of cancers [18–20]. Significant overexpression of lncRNAs-CCAT2 was found in lung adenocarcinoma [21]. Nie et al. [22] reported that the lncRNA ANRIL was overexpressed in NSCLC patient tissues and associated with advanced “tumor node metastasis (TNM)” subsets, tumor size, and prognosis. Therefore, abnormalities of the expression of lncRNAs may be involved in the tumorigenesis of LC. Genetic variants in lncRNAs could be a biomarker for the prediction of cancer susceptibility in humans. Liu et al. [23] found that lncRNAs-MALAT1_rs619586 was associated with decreased hepatocellular carcinoma risk. LncRNAs-HOTAIR_rs12826786 in strong linkage disequilibrium with rs1899663 (r^2^ = 1) was associated with the risk of gastric cardia adenocarcinoma [24]. However, their definitive roles in cancer development and progression remain largely unclear.

The H19 lncRNA gene does not encode a protein, but an oncofetal RNA [25, 26]. Deregulation of oncofetal RNA plays a critical role in tumorigenesis [26]. Accumulating evidence suggests that loss of imprinting and deregulation of the H19 gene are associated with human cancer, and its overexpression is a frequent event in lung cancer development [27, 28]. H19 is abnormally expressed in many types of cancers, including gastric [29], liver [30], colorectal [31], bladder [32], and pancreatic cancer [33], and increases the tumorigenic properties of tumor cells [34–37]. In addition, studies have shown that H19 enhances invasion and migration of pancreatic ductal adenocarcinoma cells by decreasing let-7 and subsequently increasing the HMGA2-mediated epithelial-mesenchymal transition (EMT) [33]. Barsyte-Lovejoy et al. [34] found that the knockdown of H19 inhibited colony formation and anchorage-independent growth in lung cancer cells. Other studies have reported that H19 could be induced under hypoxic stress through the p53/HIF1-α pathway. Moreover, the knockdown of H19 could significantly suppress hypoxia-induced cancer cell proliferation in vivo [36]. Furthermore, high expression of H19 was positively associated with advanced TNM stage and was a predictor of overall survival (OS) in gastric cancer patients [38, 39]. Studies have shown that the H19_rs2107425 SNP was related to the susceptibility of bladder cancer, and showed a significant correlation with LC susceptibility (*P* = 0.02, age under 50 years) [40, 41]. However, Riaz et al. [42] found that H19_rs2107425 did not alter H19 mRNA expression in breast cancer. Yang et al. [43] reported that the variant H19 genotypes (CT + TT rs217727, CT + TT rs2839698) were correlated with an increased risk of gastric cancer (*P* = 0.040, *P* = 0.033), and the CT and TT genotypes in rs2839698 were also related to higher H19 mRNA levels in serum. In contrast, the rs217727 polymorphism did not affect the H19 mRNA level. To the best of our knowledge, the role of the H19_rs217727 polymorphism in LC susceptibility is still unknown in the Chinese population. Accordingly, we investigated whether this polymorphism was associated with the risk of LC in the Chinese population.

In this study, the A/A genotype of H19_rs217727 was significantly higher in the LC patients than in the controls (*P* = 0.006). In particular, there was a significantly increased risk of squamous cells carcinoma (*P* = 0.004) and adenocarcinoma (*P* = 0.045). However, when the combined A/G + A/A genotypes were compared with the wild-type G/G genotype, there was no significant difference. Therefore, the G allele may be a protective factor and people who carry this allele may be less likely to develop lung cancer. However, the present research was limited with respect to geographical variation, nation, and sample size. These factors may greatly affect the accuracy of this experiment. Additional studies that encompass more geographical regions, additional ethnic groups, and larger sample size should be performed. Although all subjects were enrolled from only two hospitals and selection bias could not be avoided, the genotype distribution of the controls in our study did accord with the HWE. Additional studies are also necessary to understand the mechanism by which the rs217727 SNP affects H19 mRNA expression, alters the translational efficiency, or leads to alterations in the H19 structure in LC.

## Conclusions

In the current study, we found that the H19_rs217727 polymorphism plays a crucial role in the risk of LC in a Chinese population. Larger population-based studies are required to confirm the relationship between H19 expression levels and the susceptibility to LC. H19_rs217727 SNPs may be potential clinical markers for predicting the risk of LC.

## Abbreviations

AD: Adenocarcinoma
CI: Confidence interval
DMR: Differentially methylated regions
HWE: Hardy–Weinberg equilibrium
LC: Lung cancer
LD: Linkage disequilibrium
lncRNA: Long non-coding RNA
MAF: Minor allele frequency
OR: Odds ratio
SCC: Squamous cell carcinoma
SCLC: Small cell lung cancer
SD: Standard derivation
SNPs: Single nucleotide polymorphisms
